# A network analysis of dissemination and implementation research expertise across a university: Central actors and expertise clusters

**DOI:** 10.1017/cts.2022.8

**Published:** 2022-03-07

**Authors:** Reza Yousefi Nooraie, Gretchen Roman, Kevin Fiscella, James M. McMahon, Elissa Orlando, Nancy M. Bennett

**Affiliations:** 1 Department of Public Health Sciences, University of Rochester, Rochester, New York, USA; 2 Department of Family Medicine, University of Rochester, Rochester, New York, USA; 3 School of Nursing, University of Rochester, Rochester, New York, USA; 4 Clinical & Translational Science Institute, University of Rochester School of Medicine and Dentistry, Rochester, New York, USA; 5 Department of Medicine, University of Rochester, Rochester, New York, USA

**Keywords:** Capacity Building, Clinical & Translational Science Award (CTSA), Dissemination & Implementation Science, Program Assessment, Social Network Analysis

## Abstract

**Background:**

Although dissemination and implementation (D&I) science is a growing field, many health researchers with relevant D&I expertise do not self-identify as D&I researchers. The goal of this work was to analyze the distribution, clustering, and recognition of D&I expertise in an academic institution.

**Methods:**

A snowball survey was administered to investigators at University of Rochester with experience and/or interest in D&I research. The respondents were asked to identify their level of D&I expertise and to nominate others who were experienced and/or active in D&I research. We used social network analysis to examine nomination networks.

**Results:**

Sixty-eight participants provided information about their D&I expertise. Thirty-eight percent of the survey respondents self-identified as D&I researchers, 24% as conducting D&I under different labels, and 38% were familiar with D&I concepts. D&I researchers were, on average, the most central actors in the network (nominated most by other survey participants) and had the highest within-group density, indicating wide recognition by colleagues and among themselves. Researchers who applied D&I under different labels had the highest within-group reciprocity (25%), and the highest between-group reciprocity (29%) with researchers familiar with D&I. Participants significantly tended to nominate peers within their departments and within their expertise categories.

**Conclusions:**

Identifying and engaging unrecognized clusters of expertise related to D&I research may provide opportunities for mutual learning and dialog and will be critical to bridging across departmental and topic area silos and building capacity for D&I in academic settings.

## Introduction

“Quality improvement,” “improvement science,” “implementation science,” and “knowledge translation” are a few terms used, often interchangeably, to study how to support and integrate the use of evidence in health practice and policy and to address barriers to effective implementation of innovations [1-3]. Tyler and Glasgow (2021) identify “quality improvement,” “improvement science,” and “implementation science” as different sectors of delivery science. While these sectors may have different starting points, end points, and reference frames, they use similar strategies, render related outcomes, and share the common goal of improving health outcomes [4]. “Quality improvement” within healthcare systems adopts systems thinking from fields outside of healthcare [2-4]. While “quality improvement” activities are focused on addressing problems in a system, they can also be generalized to broader contexts through “improvement science” approaches. Similarly, “implementation science” focuses on methods that promote integration of the evidence into routine practice, aiming to produce generalizable solutions and insights [5,6]. Rooted in different disciplinary and geographical traditions, “knowledge” has been developed as a dynamic and iterative process of synthesis, dissemination, and application of research findings in healthcare [7,8]. All of these identified fields have the common goal of effectively integrating the scientific evidence into healthcare practice for improved patient and community outcomes. Many are still in the early stages of development [1]; hence, the commonalities and differences of these fields are not well understood.

Awareness of dissemination and implementation (D&I) science is growing. Yet, many health researchers with content or methodological expertise related to the multidisciplinary D&I science or who use novel D&I methods in their research do not self-identify as D&I scientists [9]. There are many reasons why this expertise is not fully recognized by the broader research community. The variety of terms within the respective fields used to define D&I science, access to available training, and formulation of research partnerships have been identified as perceived barriers to engaging in D&I research [5]. Over 100 terms have been identified to describe “knowledge translation” research [1,8] and at least 111 D&I models in health research and practice have been compiled [10]. While some terms like implementation, adoption, dissemination, and complex interventions were found to be significant differentiators predominantly used in D&I and “knowledge translation,” they offer little value to defining the field. Additionally, scholars have criticized the development of new variations of essential “quality improvement” methods as pseudo-innovation [7,11]. The field of “quality improvement” also has tried to consolidate taxonomies and create a unified framework; however, there has been little success in efforts to reach a synthesis [12,13].

There is a widespread call to action in response to the need for building individual and team D&I skills, as well as, organizational D&I research capacity [14,15]. Efforts to align foundational research training needs for individuals at multiple career stages while being responsive to advances in the field has been a challenge across multiple popular D&I science training programs [16]. Thus, scholars have tried to develop educational competencies for D&I research that may include a long list of potential competencies for various levels of advancement in D&I research [15,17] and overlap with many different fields of research. Arguably, many health researchers maintain many of the proposed competencies, however do not self-identify as D&I researchers [9]. On the other hand, many researchers who might have been focusing on implementation of innovations from alternative traditions (such as “quality improvement” and “community-based participatory research”) might also not consider themselves as D&I researchers based on these definitions or define D&I expertise locally or institutionally in ways that may not fully conform to the mainstream definitions of D&I.

According to the transactive memory theory, in a closely connected community, knowledge is encoded, stored, and retrieved in a system shared by different members who enjoy a collective memory of where to find certain knowledge by communicating with each other [18]. It is reasonable to assume that health researchers in an academic institution develop a general awareness about with whom the D&I and its related skillsets lie. On the other hand, researchers tend to form invisible communities of research collaboration based on shared interests and worldviews that may cross traditional institutional boundaries. These “invisible colleges [19],” “invisible communities [20],” or “epistemic communities [21]” are critical in knowledge creation and formation of emerging research fields [22]. Network analysis provides a suitable methodological perspective to assess the composition of these invisible communities [23] that are formed based on shared interest and the dynamics of expertise nomination in transactive memory systems [24].

This paper responds to the need of Clinical & Translational Science Award (CTSA) programs to further define and develop adapted solutions for what constitutes a D&I expert and to build capacity in D&I science and practice in the CTSA context. In 2020, the University of Rochester, Clinical and Translational Science Institute (UR-CTSI) established the Equity-Focused Dissemination and Implementation (EQ-DI) Function with the goal of building capacity for the science and practice of D&I with an emphasis on health equity. The charge of the EQ-DI Function is to foster a collaborative environment of mutual support and provide on-the-job training opportunities, as well as to build individual and team skills and organizational capacity for D&I research. The EQ-DI Function provides methodological consultations to researchers considering the addition of D&I into grant proposals, conducts webinar series involving external and internal experts, introduces D&I and its best practices to the clinical community, supports planning and developmental initiatives through pipeline-to-pilot mini grants, and builds capacity for incorporation of D&I into medical and nursing training. In order to foster an inclusive environment and to facilitate mutual learning of D&I science, the EQ-DI Function conducted a study of the distribution of D&I expertise within the UR and its reflection on the recognition of such expertise among peer scholars in the institution. We sought to understand better the local culture of D&I and the possible existence of invisible communities of investigators based on shared understandings of D&I research.

## Material and Methods

Because this analysis was conducted as a program assessment for the UR-CTSI, it was deemed exempt by the UR Research Subject Review Board (STUDY00006411).

### Snowball Approach

We administered a snowball survey to faculty members at the UR School of Medicine and Dentistry with experience and/or interest in D&I research. The survey was a part of a capacity building activity for the newly established EQ-DI Function at UR-CTSI. In January 2020, 46 investigators who were known by the leadership of CTSI as interested or active in D&I research participated in a meeting hosted by the EQ-DI Function to help plan for the future activities. Subsequent to this meeting, we invited these 46 individuals to participate in the first round of the snowball survey. Each respondent could nominate others of whom he/she was aware who had expertise and/or conducted activities related to D&I research, regardless of actual collaboration. In four subsequent rounds, we sent invitations to individuals nominated in previous rounds and sent two follow-up invitations to nonrespondents.

In addition to nominating other investigators who were experienced and/or interested in D&I research, the respondents were asked to self-identify their own level of D&I expertise, according to the following categories: (1) “I have done relevant research using D&I theories, models, and tools,” (2) “I have done relevant research, but under different labels,” (3) “I am familiar with the concepts and literature, but have not applied them,” and (4) “I am not familiar with these concepts.” Individuals who selected “I am not familiar with these concepts” or who were not part-time or full-time faculty members at the UR School of Medicine and Dentistry were excluded from the network analysis.

### Social Network Analysis

**Fig. 1.**
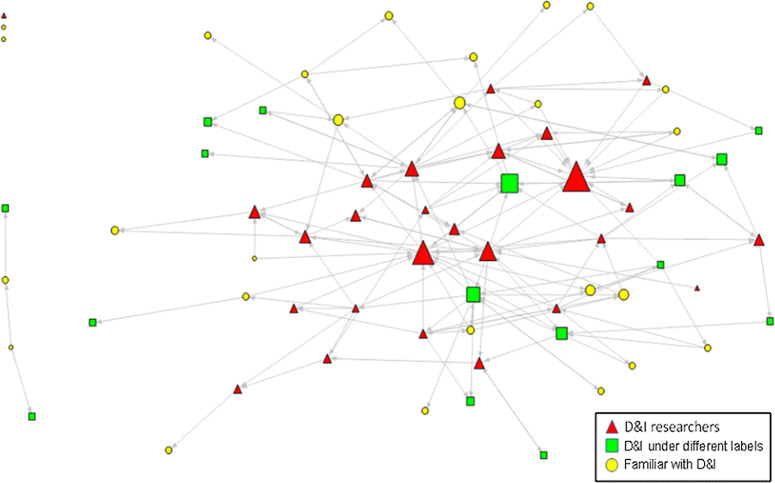
The nomination network of dissemination and implementation (D&I) expertise. The node size is proportional to in-degree centrality.

We transformed the nomination data into a matrix indicating who nominated whom, with all included individuals forming both rows and columns. If person A nominated person B, the corresponding cell in the matrix would indicate 1. We developed a nomination network map, in which the individuals were indicated as nodes and nominations as directed arrows (Fig. 1). Respondents to the snowball survey were divided based on their self-identified level of expertise. Further explained in Table 1, density and reciprocity of the nomination network and centrality of network actors were calculated as structural indicators of the network [25,26]. Within- and between-group density and reciprocity were calculated to show the dynamics of connections within and across expertise groups, respectively.

**Table 1.** Network analysis metrics

Metric	Definition
Density	The proportion of all possible relations that exist. We calculated density overall, and for within- and between-group relations (based on expertise level).
Reciprocity	The proportion of relations that are bidirectional. If A nominates B, then B also nominates A. We calculated reciprocity overall, and for within- and between-group relations (based on expertise level).
In-degree centrality	The number of incoming relations (received nominations) for each individual; an indicator of popularity.
Core-periphery analysis	An iterative process of assigning network actors to two blocks: a dense core and a sparsely connected periphery. The goodness of fit is measured by the correlation coefficient of the observed core and peripheral block assignments with an ideal matrix of the same size, showing connections between all core members and no connections between peripheral members.

We analyzed for the core–periphery pattern (Table 1) to assess the extent to which the nomination network would resemble a social structure with a densely connected core and a sparsely connected periphery. We also developed a Quadratic Assignment Procedure (QAP) regression model [27] to predict nomination ties among pairs of participants by being in the same department (i.e., nominator and nominated both are in the same department), being in the same D&I expertise category, and the difference between the D&I expertise of nominated and nominator (i.e., less experts to nominate more experts). The QAP model addressed the dependency of network ties by applying 1000 permutations of matrices. The network analysis was conducted using version 6.7 of Ucinet for Windows software [28].

## Results

### Participants

We conducted the snowball process for five rounds. At first round, from our initial list of investigators (n = 46), 22 responded. At the second round, we contacted 44 individuals including newly nominated individuals, as well as the initial contacts who did not respond. We subsequently contacted 42, 32, and 11 individuals at third, fourth, and fifth rounds, respectively. At the last round, out of 11 contacted individuals, 5 responded and only identified already nominated peers. So, we stopped the survey, as we reached information redundancy. After five rounds of snowball survey, we achieved an overall 76.4% survey response with 68 participants providing information about their D&I expertise and nominating a total of 89 investigators.

Forty percent of the survey respondents were male. The Department of Medicine (56%), School of Nursing (15%), Department of Public Health Sciences (10%), and CTSI (7%) were the most commonly affiliated departments. Twenty-six (38%) of the survey respondents who described their expertise as “I have done relevant research using D&I theories, models, and tools” were labeled as “D&I researchers.” Sixteen (24%) selected “I have done relevant research, but under different labels” and were designated as the researchers that applied “D&I under different labels.” Twenty-six (38%) selected “I am familiar with the concepts and literature but have not applied them” were noted to be “familiar with D&I” (Table 2). Zero (0%) answered “I am not familiar with these concepts.”

**Table 2.** Survey respondents’ self-identified level of dissemination and implementation (D&I) expertise

Levels of identified D&I expertise	Assigned designation	Prevalence
“I have done relevant research using D&I theories, models, and tools”	D&I researchers	38%
“I have done relevant research, but under different labels”	D&I under different labels	24%
“I am familiar with the concepts and literature, but have not applied them”	familiar with D&I	38%

### Characteristics of the Nomination Network

Overall, the nomination network revealed 4% density and 11% reciprocity (Fig. 1). The distribution of centrality varied, and visual inspection of the overall nomination network showed a main connected component including the majority of actors (except three isolates and a cluster of four individuals) with no apparent clustering. On average, the participants nominated two (ranging from zero to nine) other investigators with experience and/or interest in D&I research; however, there were three central actors in the network who identified as *D&I researchers* (Fig. 1; larger red triangles) and were nominated by 13, 10, and 7 respondents, respectively. Analysis of variance revealed the in-degree centrality (mean ± SD) of *D&I researchers* (3.4 ± 2.9) was significantly more (*p* = 0.003) than *D&I under different labels* (2.2 ± 1.8) and those *familiar with D&I* (1.3 ± 1.0). In the core–periphery analysis, the correlation between the permuted networks and the ideal core–periphery network (with *r* = 1 showing a tightly connected core and unconnected periphery) was *r* = 0.39, indicating a moderate tendency towards core–periphery structure in the network.

### Connectivity Based on D&I Expertise

**Fig. 2.**
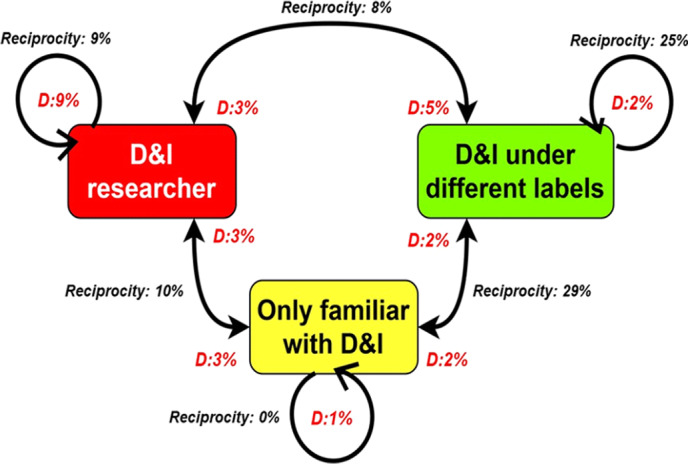
Within- and between-group density (d) and reciprocity of expertise nominations. D, density, D&I, dissemination and implementation.

Fig. 2 shows the density and reciprocity of connections within and between groups for the three participating D&I expertise levels. *D&I researchers* had the highest within-group density (9%) compared to *D&I under different labels* (2%) and those *familiar with D&I* (1%). However, *D&I under different labels* had the highest within-group reciprocity (25%) compared to *D&I researchers* (9%) and those *familiar with D&I* (0%). Between-group density nominations across the three D&I expertise groups did not show noticeable differences (3% for between *D&I researchers* and *D&I under different labels*; 3% for *D&I researchers* and those *familiar with D&I*; 5% for *D&I under different labels* and *D&I researchers*; 2% for *D&I under different labels* and those *familiar with D&I*; 3% those *familiar with D&I* and *D&I researchers*; 2% those *familiar with D&I* and *D&I under different labels*). However, **D&I under different labels** and those *familiar with D&I* had the highest between-group reciprocity (29%) compared to between *D&I researchers* and those *familiar with D&I* (10%) and between *D&I researchers* and the *D&I under different labels* (8%).

The QAP logistic regression analysis (Table 3) showed that participants were significantly more likely to nominate a peer with similar D&I expertise level (rather than a more expert peer) with an odds ratio of 1.53 ± 0.18 (*p* < 0.02). Participants also significantly tended to nominate peers in their department (compared to other departments), with an odds ratio of 1.85 ± 0.25 (*p* < 0.02). Both these findings supported the existence of localized communities of expertise.

**Table 3.** The QAP logistic regression to predict nominations

	Odds ratio (SD)	*p*-Value
Difference in D&I expertise (less experts nominating more experts)	1.04 (0.08)	0.29
Same D&I expertise levels of nominator and nominated	1.53 (0.18)	**0.015**
Nominator and nominated in the same department	1.85 (0.25)	**0.02**
Intercept	0.02 (0.53)	**0.002**

The bold values are statistically significant at *p* = 0.05.

1000 permutations, Likelihood ratio: −675, *p* = 0.007.

D&I, dissemination & implementation; QAP, quadratic assignment procedure; SD, standard deviation.

## Discussion

The objective of this work was to analyze the distribution of D&I expertise within our institution. We found that nomination network analysis was able to provide insights into the distribution of expertise and informal clusters in the institution. While 62% of nominated individuals self-identified as either using D&I theories, models, and tools or applied D&I science under different labels in their research, there was low interconnectedness and mutual recognition of D&I expertise across the entire group. Those who self-identified as *D&I researchers* were nominated most often by others (as reflected by their in-degree centrality), had the greatest percentage of within-group connections (i.e., density), and formed a relatively central core in the network. This indicates a known sense of who maintains D&I expertise among colleagues within the group of D&I researchers; however, the within-group interconnectedness was still relatively low. Those conducting *D&I under different labels* had the greatest mutual nomination of each other (i.e., within-group reciprocity) and with the group who were only *familiar with D&I*, hence were thought to be an unrecognized expertise cluster within the network. The regression analysis showed that the participants were more likely to nominate within their expertise category and within their own department. This high reciprocity of nominations and expertise and departmental homophily imply that in this institution, researchers may recognize D&I expertise based on factors that do not necessarily conform to the mainstream/traditional definitions of D&I science and could indicate the existence of a localized culture of D&I research (perhaps more along the lines of “quality improvement” and community-based participatory research).

Potential challenges to the adoption of D&I science by clinical researchers are their lack of familiarity with its principles and terminologies, existence of other parallel scientific traditions (such as “quality improvement” and “evidence-based medicine”), and potential lack of familiarity and recognition of the need for a specific scientific D&I dedicated field [29,30]. In a national survey of CTSA hubs about their support of and barriers to integrating D&I science activities, Dolor et al. [29] found that roughly half of CTSA hubs provide direct support to D&I; and, inadequate D&I science workforce and lack of understanding of D&I science were identified by the majority of respondents as barriers to capacity building for D&I science. Many of the challenges related to D&I of clinical innovations have been traditionally studied using methods borrowed from other fields, such as education research to develop and test training programs for clinicians or by adopting models from “quality improvement” research [2-4].

Recognizing the existence of such alternative efforts and initiatives to investigate barriers to and improve implementation will be critical in facilitating the integration of D&I theoretical frameworks, tools, and outcomes into existing clinical and organizational research traditions. D&I researchers can play critical translational boundary crossing roles on research teams to facilitate dialog and collaborations between various disciplines [31]. This study showed that already established cultures and communities exist within our institution that fall within the larger umbrella of D&I science. We need to develop solutions to enrich these cultures, rather than rejecting and replacing them with orthodox D&I mindsets. This has several implications for D&I scientists, not only as domain experts, but also as boundary crossers, team players, systems thinkers, process innovators, and skilled communicators [32]. The inclusive recognition of existing traditions will provide opportunities for mutual learning and acceptance.

Brownson et al. [33] shared seven challenges encountered upon building organizational D&I research capacity at academic institutions: lack of awareness about D&I, the broad scope of D&I science, the need for resources, the need of academic leadership and networking, the need to balance consultation and time for research, the need to move beyond the walls of academia, and the need to build greater focus on evaluation. Findings from our study suggest an eighth challenge: building the D&I culture upon existing institutional expertise. Researchers can use various approaches to gain a better understanding of local cultures and traditions, including quantitative and qualitative studies. These localized communities form “invisible colleges [19]” that represent informal clusters of investigators. Identifying these “invisible colleges” is critical in addressing inherently complex and transdisciplinary fields, such as implementation science [34]. Network analysis provides opportunities to study cultures and communities as social and relational phenomena, and is a useful perspective to discover those unrecognized clusters of expertise [20].

The UR-CTSI EQ-DI Function was developed to build clinical and translational researchers’ capacity for engaging in D&I research and to promote D&I as a critical component of everyday scientific conversations. We were aware of the limited expertise in this context, but the findings of this study provided insight into the receptiveness toward novel D&I innovations. We believe that it is critical to recognize alternative traditions and definitions and to facilitate dialog to bridge organizational and disciplinary silos. These findings will inform our future activities by providing insights into the breadth and connectivity within- and across-expertise clusters, as well as, identifying central actors among the various D&I expertise levels, many of whom are in leadership positions. Central actors, even if they do not fully conform to our classical definitions of D&I science, are potential opinion leaders who may assist in the diffusion of D&I culture across silos.

There were a few limitations to this study. We used a nomination method to identify D&I expertise in our institution. As a result, our findings may be biased towards those more familiar with D&I, as we limited our assessment to nominating individuals with experience and/or interest in D&I research and did not survey all research faculty to understand the distribution of D&I expertise across the entire institution. An institution-wide survey of D&I expertise may identify additional investigators and other less recognized clusters that we might have missed. However, we believe that our targeted method efficiently identified active researchers in D&I (and related fields). This is especially suitable in our institution in which D&I is less known by the majority of health and clinical researchers. Other CTSA institutions can decide the best approach based on the prior knowledge of D&I expertise and the availability of resources to conduct institution-wide surveys. In addition, we did not further explore the diversity of expertise and experience with D&I, which could ideally be explored in a qualitative assessment. Future directions of this research may involve a longitudinal assessment of the network of D&I researchers and their expertise after enacting organizational capacity building efforts to bolster the network interconnectedness, such as the webinar series and other participatory activities.

In summary, the size and connectivity of the cluster of investigators doing D&I research under different labels demonstrates that researchers in this academic institution recognize D&I expertise beyond mainstream definitions of D&I. When building organizational capacity for D&I, it is important to recognize the existence of local definitions and cultures of D&I research. A broader acceptance and acknowledgement of local definitions and cultures may help reduce perceived barriers and facilitate the adoption and proliferation of D&I methods. Identification of influential actors across various expertise categories can be pivotal to promoting dialog and connectivity across the entire network. Bridging silos will inspire individuals who possess advanced D&I skills to share their expertise with individuals and teams to build organizational capacity for D&I.
